# Correction: Understanding patient and family experiences of critical care in Bangladesh and India: What are the priority actions to promote person-centred care?

**DOI:** 10.1371/journal.pgph.0003894

**Published:** 2024-10-23

**Authors:** Rebecca Inglis, Meghan Leaver, Christopher Pell, Suma Ahmad, Shamima Akter, Fakrul Ibne Amir Bhuia, Mumnoon Ansary, Sidharth B. S., Momtaz Begum, Shishir Ranjan Chakraborty, Hasnat Chowdhury, Mohammed Abdur Rahman Chowdhury, Putul Deb, Nazmin Akhter Farzana, Aniruddha Ghose, Mohammad Harun Or Roshid, Md. Rezaul Hoque Tipu, Sakib Hosain, Md. Mozaffer Hossain, Mohammad Moinul Islam, Bharath Kumar Tirupakuzhi Vijayaraghavan, Mohammad Mohsin, Manisha Mund, Shamema Nasrin, Ranjan Kumar Nath, Subhasish Nayak, Nibedita Pani, Shohel Ahmmad Sarker, Arjen Dondorp, Swagata Tripathy, Md. Abul Faiz

## Notice of Republication

An incorrect version of data availability statement was published in error. The article was republished on October 15, 2024, to correct this error. Please download the article again to view the corrected version.
