# Supplementary material for: Understanding patient and family experiences of critical care in Bangladesh and India: What are the priority actions to promote person-centred care?
Source: PLOS Glob Public Health. 2024 Jun 28;4(6):e0003372. doi: 10.1371/journal.pgph.0003372 (PMC11213345; doi:10.1371/journal.pgph.0003372)
Supplement: S1 Text — (PDF) [file pgph.0003372.s002.pdf]

Note - not all topic areas will be covered in all interviews and certain topics will be expanded on further at the discretion of the research team. They will also be adapted as required during the course of the study.

INTERVIEW GUIDE FOR PATIENT FAMILIES

Instructions:

- Conduct the interview in a quiet, private location

- Obtain written informed consent before starting the interview

- If consent is given, audio record the interview

- This interview guide is a prompt to be used in a flexible manner. You do not need to follow the order of topics below; follow the responses/flow of the conversation. Use a flexible approach and probe as necessary: add extra questions depending on the responses

Opening comments:

Many thanks for agreeing to talk to us – it is very kind of you to make time. I know this must be a very difficult and upsetting time for you at the moment.

The interview will probably take between 30 and 40 minutes.

If you wish to stop the interview at any point that is totally fine – do not worry at all.

Everything you tell us in the interview will be totally confidential. If there are any questions you don't feel comfortable to answer that is totally fine too – just say 'I don't want to answer that' and we will move on to the next question.

Our standard procedure is to make an audio recording of interviews, so we have an accurate record of what you have said. Again, the audio file is entirely confidential and will not be shared with anyone. If you prefer not to be recorded, I can just take notes instead.

Finally, it is important to say this will not affect the care that your loved one receives. Also it is not a test and we are not trying to catch you out. We hope to learn from your experience.

Note - not all topic areas will be covered in all interviews and certain topics will be expanded on further at the discretion of the research team. They will also be adapted as required during the course of the study.

Section 1 – Introduction:

- What do you do for a living?
- Do you mind telling me how old you are?
- What does your loved one do for a living?
- How long has your loved one been on ICU?

Section 2 – Experience of ICU:

- What did you know about ICU **before** your relative was admitted? Had you ever been to one before? Did you know what ICUs do? What did you think?
- Please tell me everything you can remember about the day when your loved one first arrived on ICU. Tell me in as much detail as possible.

  Prompt if needed –

  Who did you talk to?
  How did it feel to be on ICU?
  Did anyone explain what you should and shouldn't do?
  How did the machines make you feel?
  What about all the noises?
  What did they find scary?
  What did they find reassuring?
  What was it like for your loved one?
- What are the visiting hours on ICU?
- Have you been able to help provide care for your relative?
- What have your experiences been like since that first day?
- What has the experience been like for your loved one?
- What has anything made your / their experience of ICU better? Please explain in as much detail as possible.
- What are the most difficult things about being on ICU?
- What is most important to you about the care that your loved one receives?

Section 3 – Communication:

- How often do you speak to the nurses on ICU?

- How often do you speak to the doctors?
- Have they explained what the diagnosis is for your loved one?
- Have they explained what treatments he / she is receiving?
- Have they explained what they think is likely to happen over the next few days or weeks?
- What is worrying you most at the moment?
- Have you been able to raise those worries with the staff?
- Have you felt listened to?

Section 4 – Conclusion:

- What have been the hardest parts of your experience on ICU?
- What has helped you to cope better?
- If you could change something on ICU that you think would make a real difference to patients and their families, what would it be?
- Is there anything else you would like to add?

INTERVIEW GUIDE FOR JUNIOR DOCTORS

Instructions:

- Conduct the interview in a quiet, private location

- Obtain written informed consent before starting the interview

- If consent is given, audio record the interview

- This interview guide is a prompt to be used in a flexible manner. You do not need to follow the order of topics below; follow the responses/flow of the conversation. Use a flexible approach and probe as necessary: add extra questions depending on the responses

Opening comments:

Introduce yourself first, then mention the following:

Many thanks for agreeing to talk to us – it is very kind of you to make time.

The interview will probably take between 40 and 50 minutes.

If you need to go at any time because you get called away to a patient that is totally fine – do not worry at all.

As with everything we are doing, everything you tell us in the interview will be anonymous.

Finally, it is important to say this is not test and we are not trying to catch you out. There aren't right or wrong answers to these questions. We hope to learn from your experience and your thoughts.

Section 1 – Introduction and role related information:

- How long have you been working in intensive care?
- Please can you briefly describe the ICU training you have received so far?
- Can you describe your role within the ICU team? What are your responsibilities?
- Do you enjoy working on ICU? Why?
- Do you plan to continue doing ICU once you complete your training? Can you explain why?
- Have you ever worked on a private ICU? Please describe the main differences. Are there any things from the private sector you thing would be helpful to start here?
- What sources of information do you use when you want to learn new information about ICU?

  [*If they say 'the internet' please ask which sites; if they say 'medical journals' please ask which and how do they find what they want to read (eg pub med search or something else); if they say books, please ask which; if they say other people, please ask who, how and when*).

Section 2 – Barriers and facilitators:

EXPERIENCE OF ICU SHIFTS

- We have seen that ICU is often very busy for the duty doctor. What aspects of your shift do you find hardest?
- What things help to make your work easier?
- Do you have any suggestions of things that could be changed to make your shifts easier?
- During your shift, how do you prioritise which tasks to do first?
- How do you think night shifts could be made safer?

ICU ADMISSIONS

- Who decides which patients are admitted to ICU?

- When you are deciding if a patient should be admitted to ICU, what factors do you take into consideration?

- Please describe the process from a patient being referred to ICU, to the decision being made about whether or not to admit them, to potentially being admitted to ICU.

- Are there any patients who you don't think should be admitted to ICU? Please explain why.

VENTILATION

- Are you able to operate the ventilators? Which aspects are you able to do? Which bits do you need a more senior doctor to help you with? Which bits do the nurses do?

- When you start a patient on a ventilator, do you generally set specific alarm boundaries or just leave them as they are? If you set the alarms for each patient, which ones do you set? How do you decide on how to set them?

- Who decides how much analgesia and how much sedation to give patients on the ventilator? What level of sedation are you targeting in patients?

- When an alarm rings on the ventilator, who do you expect to respond to it? The doctors or the nurses?

- Please can you describe how patients are weaned off the ventilator on this ICU?

HANDOVER

- When you start your shift, how do you get familiar with the patients you are looking after?
  [*If they don't understand, ask* 'is it mainly handover from the previous shift's doctor, from the nurses or from the patient notes?']

- At the end of your shift, please describe how you handover to the next doctor on shift.

- Have you ever been taught about how you should handover between shifts?

PAIN MANAGEMENT AND REHABILITATION

- Please describe how you assess and treat pain on ICU.
- We know that encouraging a patient to move as soon as possible is important to improve their recovery. Please can you describe what patient mobilization / physiotherapy happens on ICU?
- Who decides when to start mobilization and what to do? The doctors or the nurses?

PATIENT FAMILIES

- How do you find communicating with patient's families? What are the challenges?
- What do you think are the best aspects about how your ICU team communicates with families?
- Are there any ways communication with families could be improved?

EQUIPMENT

- Do you have any challenges with the machines on ICU? Can you describe them in detail?
- Which machines are easiest to use? Why? Please give lots of detail.

PATIENT EXPERIENCE

- What do you think the experience is like for people who are admitted to ICU as patients? [Please get them to answer this in as much detail as possible).
- What things can make their experience better?
- What things can make their experience worse?
- How can we improve the experience for families who have loved ones on ICU? Please describe everything you can think of.

VERBAL COMMUNICATION

- What do you think communication is like between the doctors and the nurses? Please give examples if you can.
- Do you have any ideas about how to improve communication between the doctors and the nurses?
- What do you think the communication is like with patients and their families on this ICU?
- Do you have any ideas about how to improve communication between the doctors and the patient's families?
- Have you ever had training about how to communicate better?

WRITTEN COMMUNUCATION

- Please can you describe the main things that you write in the patient's medical notes each day?
- When you started ICU, what were you taught about writing in the notes? What are the official expectations about writing in the medical notes?
- Has there ever been any confusion about what medications the patient has been prescribed? For example, has there ever been a copying error between the medications you have requested and the medications that get copied by the nurses onto the treatment chart?
- When a patient is discharged from ICU, how does the receiving team know about what has been happening on ICU?

Section 4 – Conclusion:

- What do you enjoy most about your job?
- What do you enjoy least about your job?
- What aspects of patient care do you think your ICU does really well?
- What aspects of patient care do you think your ICU does less well? Why?

- In your view, what does 'high quality care on ICU' actually mean? What does it involve? Please describe all the things that contribute to high quality care on ICU.

- If you could change something on ICU that you think would make a real difference to the quality of care, what would it be?

- Is there anything else you would like to add?

At the end of the interview, thank them for their time and let them know if they think of anything they would like to add they can tell you any time.

INTERVIEW GUIDE FOR JUNIOR NURSES

Instructions:

- Conduct the interview in a quiet, private location

- Obtain written informed consent before starting the interview

- If consent is given, audio record the interview

- This interview guide is a prompt to be used in a flexible manner. You do not need to follow the order of topics below; follow the responses/flow of the conversation. Use a flexible approach and probe as necessary: add extra questions depending on the responses

Opening comments:

Introduce yourself first, then mention the following:

Many thanks for agreeing to talk to us – it is very kind of you to make time.

The interview will probably take between 40 and 50 minutes.

If you need to go at any time because you get called away to a patient that is totally fine – do not worry at all.

As with everything we are doing, everything you tell us in the interview will be totally confidential.

Finally, it is important to say this is not test and we are not trying to catch you out. There aren't right or wrong answers to these questions. We hope to learn from your experience and your thoughts.

Section 1 – Introduction and role related information:

- How many years have you been working in intensive care?
- Have you always worked in this ICU?
- Have you had training in critical care nursing or just learnt on the job?
- Please can you briefly describe the ICU training you have received?
- Did you find that your training prepared you well for working as a nurse? Please give examples if you can.
- What wards have you worked on since qualifying as a nurse?
- Why did you choose ICU?
- Do you enjoy working on ICU? Why?
- Can you describe your role within the ICU team?
- Where do you learn new information from about ICU?

Section 2 – Barriers and facilitators:

VENTILATION

- Are you able to operate the ventilators? Which aspects are you able to do? Which bits do you need a more senior nurse to help you with? Which bits do the doctors do?
- How do you find caring for patients on the ventilator? Which bits are difficult and which bits are easy about caring for patients on the ventilator?
- What are the daily tasks that the nurses need to do for a patient on a ventilator?
- Do the nurses or the doctors turn the oxygen up and down on the ventilator?
- When an alarm goes off on the ventilator, who do you expect to respond to it? The doctors or the nurses?
- What are the common problems that make the alarm go off on the ventilator?
    - [For each different alarm] If that happens, what do you do? Please describe in detail.
    - Which ones can the nurses solve by themselves?
    - Which ones need a doctor?

- What if an alarm goes off when there are no nurses in the room?

- How much time do they need to spend at the bedside of a patient on a ventilator?

PATIENT FAMILIES

- How much time are patient families allowed to spend on ICU?
- What tasks are patient's families able to do for the patient while on ICU?
- When a new patient arrives on ICU what do you explain to their family about ICU? Please pretend I am a the relative of a new patient and tell me everything you normally would.
- What do you tell them about preparing food for the patient?
- Do you find the patient's families are helpful to you in delivering patient care?
- Are there ever any problems with the patient's families helping?

PATIENT EXPERIENCE

- What do you think the experience is like for people who are admitted to ICU as patients? [Please get them to answer this in as much detail as possible).
- What things can make their experience better?
- What things can make their experience worse?
- How can we improve the experience for families who have loved ones on ICU? Please describe everything you can think of.
- Please tell me about patient mobilisation and physiotherapy on your ICU. When does it usually start? Who decides when it can start? Who is in charge? What does it involve?

COMMUNUCATION

- What do you think communication is like between the doctors and the nurses? Please give examples if you can.

- Are you usually clear about what the doctor's plan is for the patient? How do you find out their plan?
- Do you have any ideas about how to improve communication between the doctors and the nurses?
- When there is an emergency on the ICU, are you usually clear about what is happening or do you just do what the doctor asks you to do?
- Have you ever had training about how to communicate better?

Section 3 – Conclusion:

- What do you enjoy most about your job?
- What do you enjoy least about your job?
- What aspects of patient care do you think your ICU does really well?
- What aspects of patient care do you think your ICU does less well? Why?
- In your view, what does 'high quality care on ICU' actually mean? What does it involve?
- If you could change something on ICU that you think would make a real difference to the quality of care, what would it be?
- Is there anything else you would like to add?

At the end of the interview, thank them for their time and let them know if they think of anything they would like to add they can tell you any time.

Note - not all topic areas will be covered in all interviews and certain topics will be expanded on further at the discretion of the research team. They will also be adapted as required during the course of the study.

INTERVIEW GUIDE FOR PATIENTS

Instructions:

- Conduct the interview in a quiet, private location

- Obtain written informed consent before starting the interview

- If consent is given, audio record the interview

- This interview guide is a prompt to be used in a flexible manner. You do not need to follow the order of topics below; follow the responses/flow of the conversation. Use a flexible approach and probe as necessary: add extra questions depending on the responses

Opening comments:

Many thanks for agreeing to talk to us – it is very kind of you to make time. I know this must be a very difficult and upsetting time for you at the moment.

The interview will probably take between 30 and 40 minutes.

If you wish to stop the interview at any point that is totally fine – do not worry at all.

Everything you tell us in the interview will be totally confidential. If there are any questions you don't feel comfortable to answer that is totally fine too – just say 'I don't want to answer that' and we will move on to the next question.

Our standard procedure is to make an audio recording of interviews, so we have an accurate record of what you have said. Again, the audio file is entirely confidential and will not be shared with anyone. If you prefer not to be recorded, I can just take notes instead.

Finally, it is important to say this will not affect the care that you receive. Also it is not a test and we are not trying to catch you out. We hope to learn from your experience.

Note - not all topic areas will be covered in all interviews and certain topics will be expanded on further at the discretion of the research team. They will also be adapted as required during the course of the study.

Section 1 – Introduction:

- What do you do for a living?
- Do you mind telling me how old you are?
- How long were you on ICU?

Section 2 – Experience of ICU:

- What did you know about ICU **before** you were admitted? Had you ever been to one before? Did you know what ICUs do? What did you think about them?
- Please tell me everything you can remember about the day when you first arrived on ICU. Tell me in as much detail as possible.

  Prompt if needed –

  Who did you talk to?
  How did it feel to be on ICU?
  Did anyone explain what you should and shouldn't do?
  How did the machines make you feel?
  What about all the noises?
  What did they find scary?
  What did they find reassuring?
  What was it like for your relatives?

- What have your experiences been like since that first day?
- Were your relatives able to visit you on ICU?
- What has the experience been like for relatives?
- What has anything made your / their experience of ICU better? Please explain in as much detail as possible.
- What are the most difficult things about being on ICU?
- What is most important to you about the care that you received on ICU?

Section 3 – Communication:

- How often did you speak to the nurses on ICU?
- How often did you speak to the doctors?

- Did they explain what your diagnosis was?
- Did they explain what treatments you were receiving?
- Have they explained what they think is likely to happen over the next few days or weeks?
- What is worrying you most at the moment?
- Have you been able to raise those worries with the staff?
- Have you felt listened to?

Section 4 – Conclusion:

- What have been the hardest parts of your experience on ICU?
- What has helped you to cope better?
- If you could change something on ICU that you think would make a real difference to patient and their families, what would it be?
- Is there anything else you would like to add?

INTERVIEW GUIDE FOR SENIOR DOCTORS

Instructions:

- Conduct the interview in a quiet, private location

- Obtain written informed consent before starting the interview

- If consent is given, audio record the interview

- This interview guide is a prompt to be used in a flexible manner. You do not need to follow the order of topics below; follow the responses/flow of the conversation. Use a flexible approach and probe as necessary: add extra questions depending on the responses

Opening comments:

Many thanks for agreeing to talk to us – it is very kind of you to make time.

The interview will probably take between 40 and 50 minutes.

If you need to go at any time because you get called away to a patient that is totally fine – do not worry at all.

As with everything we are doing, everything you tell us in the interview will not have your name attached to it.

Our standard procedure is to record the audio of interviews, so we have an accurate record of what you have said. Again, the audio file will be kept confidential and will not be shared with anyone.

Finally, it is important to say this is not a knowledge test and we are not trying to catch you out. We hope to learn from your experience and opinions.

Section 1 – Introduction and role related information:

- How many years have you been working in intensive care?
- How long have you worked in this ICU?
- Do you currently work in ICU **AND** anaesthesia or just ICU? If both, how much time do you spend on each?
- How long ago did you complete your intensive care training?
- Please can you briefly describe your training pathway since medical school? What ICU training did you do and where?
- Have you ever worked on a private ICU?
    - If so, please describe the main differences between your experiences of a private ICU and this ICU.
    - Are there any things from the private sector you think would be helpful to start here? Or anything that you have started already?
    - Is there anything that you think works better here (government ICU) than there (private ICU)?
- Why did you choose a career in ICU?
- Do you enjoy working on ICU? Why?
- Can you describe your role within this ICU team?
- Can you describe the roles of the other staff working on ICU in as much detail as possible? For example, who is responsible for detecting if a patient is deteriorating? Who is responsible for updating families?

Section 2 – Barriers and Facilitators

EXPERIENCE OF ICU WORK

WARD ROUNDS

- How often will you do a ward round when you are responsible for ICU?
- Please describe how you run the ward round in as much detail as possible...

Extra questions to explore:

- What role (if any) does the nurse-in-charge play during the ward round?
- What role (if any) does the bedside nurse play during the ward round?
- What role do the junior doctors play during the ward round?
- How do you make sure you have covered all the important issues? Do you have a way to make sure you don't forget things during the ward round (for example some people use checklist or mnemonics both other people don't find these helpful)?
- How do you like your findings and management plan to be documented?

COLLABORATING WITH THE DUTY DOCTOR

- In between the days when you do a ward round, how to do you keep track of what is happening with the patients?
  - Do you use a written list or just remember what is happening?
  - How often do you meet with the duty doctor to discuss patients?
  - When you discuss the patients with the duty doctor, do you discuss every patient on ICU or only the patients that the duty doctor has a question about?
  - Do you ever phone for an update or wait for them to call you?
  - During your most recent month on duty, how many times did you have to come back into ICU from home because there was a problem that needed you to be there in person? Was this more or less than an average duty month?

- We have seen that the senior doctors on ICU are very busy. Is there anything that would make your job easier?

- We have seen that ICU work is also very busy for the junior doctor. Do you have any suggestions of things that could be changed to make their shifts easier?

- How do you think night shifts could be made safer?

TRAINING

- How do you think we can improve ICU training for junior doctors?

- How do you think we can improve ICU training for ICU nurses?

HANDOVER

- When you **start** your period of time on duty, how do you get familiar with the patients you are looking after?
  [*If they don't understand, ask* 'is it mainly handover from the previous doctor on duty, from the junior doctors or from the patient notes?']

- At the end of your period of duty, please describe how you handover to the next senior doctor.

- If you alternate days on ICU with another senior doctor, how do you handover between each from one day to the next? (eg phone call / in person / no formal process to handover etc)

- Have you ever been taught about how you should handover?

ICU ADMISSIONS

- Who decides which patients are admitted to ICU?

- When you are deciding if a patient should be admitted to ICU, what factors do you take into consideration?

- Please describe the process from a patient being referred to ICU, to the decision being made about whether or not to admit them, to potentially being admitted to ICU.

- Do you find it challenging to prioritize which patients to admit to ICU? If yes, please describe why.

- Are there any patients who you don't think should be admitted to ICU? Please explain why.

- A common scenario in ICUs everywhere is that there are not always enough beds for the patients who could benefit from ICU care. Can you think of any solutions for this?

Guidelines, protocols and checklists

On ICU, guidelines, protocols or checklists can sometimes help us to provide standardized care to patients.

- What protocols, guidelines or checklists, if any, are used on your ICU? Please tell me all the ones you can think of.

For each one they mention, ask the following questions:

  - Was it developed locally or nationally? How long ago?
  - Does it get used often? Why / why not?
  - Do you find it helpful? Why / why not?
  - Are they strictly enforced or are people free to deviate from them if they wish?
  - Are they easy to access? Is there a copy of the guideline / protocol / checklist on the ward or easily available online?

- Are there any areas where you don't currently have a guideline / protocol / checklist where you think it would be helpful to develop one?

PATIENT EXPERIENCE

- What do you think the experience is like for people who are admitted to ICU as patients? [Please get them to answer this in as much detail as possible).

- What things can make their experience better?

- What things can make their experience worse?

- How can we improve the experience for families who have loved ones on ICU? Please describe everything you can think of.

COMMUNICATION

- What do you think communication is like between the doctors and the nurses? Please give examples if you can.

- Do you have any ideas about how to improve communication between the doctors and the nurses?

- What do you think the communication is like with patients and their families on this ICU?

- Do you have any ideas about how to improve communication between the doctors and the patient's families?

- Have you ever had training about how to communicate better?

VENTILATION

- When you start a patient on a ventilator, do you generally set specific alarm limits or just leave them as they are? If you set the alarms for each patient, which ones do you set? How do you decide on how to set them?

- Who decides how much analgesia and how much sedation to give patients on the ventilator? What level of sedation (what RASS score) are you targeting in patients?

- When an alarm rings on the ventilator, who do you expect to respond to it? The doctors or the nurses?

- What level of monitoring should the ventilated patients receive?

- Please can you describe how patients are weaned off the ventilator on this ICU?

PAIN MANAGEMENT AND REHABILITATION

- Please describe how you assess and treat pain on ICU.

- We know that encouraging a patient to move as soon as possible is important to improve their recovery. Please can you describe what patient mobilization / physiotherapy happens on ICU?

- Who decides when to start mobilization and what to do? The doctors or the nurses?

PATIENT MONITORING

- How frequently do you think the patients on ICU should be monitored?

- How do you think we can improve the detection of deteriorating patients on ICU?

EQUIPMENT

- Do you have any challenges with the machines on ICU? Can you describe them in detail?

- Which machines are easiest to use? Why? Please give lots of detail.

Section 3 – Conclusion:

- What do you enjoy most about your job?
- What do you enjoy least about your job?
- What aspects of patient care do you think your ICU does really well? Please give lots of detail.
- What aspects of patient care do you think your ICU does less well? Why?
- In your view, what does 'high quality care on ICU' actually mean? What does it involve? Please describe all the things that contribute to high quality care on ICU.
- If you could change something on ICU that you think would make a real difference to the quality of care, what would it be?
- Is there anything else you would like to add?

INTERVIEW GUIDE FOR SENIOR NURSES

Instructions:

- Conduct the interview in a quiet, private location

- Obtain written informed consent before starting the interview

- If consent is given, audio record the interview

- This interview guide is a prompt to be used in a flexible manner. You do not need to follow the order of topics below; follow the responses/flow of the conversation. Use a flexible approach and probe as necessary: add extra questions depending on the responses

Opening comments:

Introduce yourself first, then mention the following:

Many thanks for agreeing to talk to us – it is very kind of you to make time.

The interview will probably take between 40 and 50 minutes.

If you need to go at any time because you get called away to a patient that is totally fine – do not worry at all.

As with everything we are doing, everything you tell us in the interview will be anonymous.

Finally, it is important to say this is not a test and we are not trying to catch you out. There aren't right or wrong answers to these questions. We hope to learn from your experience and your thoughts.

Section 1 – Introduction and role related information:

- How long have you been working in intensive care?
- Please can you briefly describe the ICU training you have received so far?
- Can you describe your role within the ICU team? What are your responsibilities?
- Do you enjoy working on ICU? Why?
- Why did you choose ICU as a specialty?
- Have you ever worked on a private ICU? Please describe the main differences. Are there any things from the private sector you thing would be helpful to start here?
- What sources of information do you use when you want to learn new information about ICU?
- Have you ever had training in management skills?

Section 2 – Barriers and facilitators:

EXPERIENCE OF ICU SHIFTS

- We have seen that ICU is often very busy for the nurses. What aspects of your shift do you find hardest?
- What things help to make your work easier?
- Do you have any suggestions of things that could be changed to make your shifts easier?
- During your shift, how do you prioritise which tasks to do first?
- How do you think night shifts could be made safer?

ICU ADMISSIONS

- Are there any patients who you don't think should be admitted to ICU? Please explain why.

VENTILATION

- Are you able to operate the ventilators? Which aspects are you able to do? Which bits do you need a doctor to help you with?
- Who decides how much analgesia and how much sedation to give patients on the ventilator?
- What level of sedation are you targeting in patients?
- When an alarm rings on the ventilator, who do you expect to respond to it? The doctors or the nurses?
- Please can you describe how patients are weaned off the ventilator on this ICU?

HANDOVER

- When you start your shift, how do you get familiar with the patients you are looking after?
  [*If they don't understand, ask '*is it mainly handover from the previous shift's nurse, from the doctors or from the patient notes?']
- At the end of your shift, please describe how you handover to the next nurse on shift.
- Have you ever been taught about how you should handover between shifts?

PAIN MANAGEMENT AND REHABILITATION

- Please describe how you assess and treat pain on ICU.
- Have you ever been taught about delirium? Do you know what it is?
- We know that encouraging a patient to move as soon as possible is important to improve their recovery. Please can you describe what patient mobilization / physiotherapy happens on ICU?
- Who decides when to start mobilization and what to do? The doctors or the nurses?
- Did you receive training in physiotherapy and mobilization?

PATIENT FAMILIES

- How much time are patient families allowed to spend on ICU?
- What tasks are patient's families able to do for the patient while on ICU?
- When a new patient arrives on ICU what do you explain to their family about ICU? Please pretend I am a the relative of a new patient and tell me everything you normally would.
- What do you tell them about preparing food for the patient?
- Do you find the patient's families are helpful to you in delivering patient care?
- Are there ever any problems with the patient's families helping?
- How do you find communicating with patient's families? What are the challenges?
- What do you think are the best aspects about how your ICU team communicates with families?
- Are there any ways communication with families could be improved?

EQUIPMENT

- Do you have any challenges with the machines on ICU? Can you describe them in detail?
- Which machines are easiest to use? Why? Please give lots of detail.

PATIENT EXPERIENCE

- What do you think the experience is like for ICU patients? [Please get them to answer this in as much detail as possible).
- What things can make their experience better?
- What things can make their experience worse?
- How can we improve the experience for the families of ICU patients? Please describe everything you can think of.

VERBAL COMMUNICATION

- What do you think communication is like between the doctors and the nurses? Please give examples if you can.
- Are you usually clear about what the doctor's plan is for the patient? How do you find out their plan?
- Do you feel confident to speak to the senior doctors if you notice a problem with the patient?
- Do you have any ideas about how to improve communication between the doctors and the nurses?
- What do you think the communication is like with patients on this ICU?
- Do you have any ideas about how to improve communication between the doctors and the patients?
- Have you ever had training about how to communicate better?

WRITTEN COMMUNUCATION

- When you started ICU, what were you taught about writing in the notes? What are the official expectations about writing in the medical notes?
- Has there ever been any confusion about what medications the patient has been prescribed? For example, has there ever been a copying error between the medications that the doctors write in the notes and the ones that get copied onto onto the treatment chart?
- When a patient is discharged from ICU, how does the receiving team know about what has been happening on ICU? Do you pass on any information to the nurses directly?

PATIENT MONITORING

- How can you tell if a patient is sick enough to need to call the doctor?

Section 4 – Conclusion:

- What do you enjoy most about your job?
- What do you enjoy least about your job?
- What aspects of patient care do you think your ICU does really well?
- What aspects of patient care do you think your ICU does less well? Why?
- In your view, what does 'high quality care on ICU' actually mean? What does it involve? Please describe all the things that contribute to high quality care on ICU.
- If you could change something on ICU that you think would make a real difference to the quality of care, what would it be?
- Is there anything else you would like to add?

At the end of the interview, thank them for their time and let them know if they think of anything they would like to add they can tell you any time.
