# Supplementary material for: Understanding patient and family experiences of critical care in Bangladesh and India: What are the priority actions to promote person-centred care?
Source: PLOS Glob Public Health. 2024 Jun 28;4(6):e0003372. doi: 10.1371/journal.pgph.0003372 (PMC11213345; doi:10.1371/journal.pgph.0003372)
Supplement: Attachment — Submitted filename: Response to reviewers.pdf [file pgph.0003372.s003.pdf]

19th April, 2024

Dear Dr. Barman,

Re: Manuscript PGPH-D-23-02475, Response to Reviewers

Thank you for giving us the opportunity to submit a revised draft of the manuscript PGPH-D-23-02475 “Understanding patient and family experiences of critical care in Bangladesh and India: what are the priority actions to promote person-centred care?” to PLOS Global Public Health.

We have incorporated the suggestions made by the reviewers. Those changes are highlighted within the manuscript. Please see below for a point-by-point response to the reviewers’ comments.

Response to Reviewers' Comments to the Authors:

- **Reviewer 1, Comment 1**

‘The findings of the study are insightful’.

Author response: Thank you.

- **Reviewer 1, Comment 2**

‘The study aimed to address three research questions, the presentation of findings for the same can be in line with these questions. Currently the broad themes identified do not clearly address any of these questions, rather a mixture of Q2 and Q3. Also, a diagrammatic representation of the themes identified pertaining to each of these questions can bring out better clarity for the reader.’

Author response: Thank you for highlighting this. We have reworded our research questions to align more closely with how they are presented in the results section and have included a diagrammatic representation of the themes generated as you suggest.

- **Reviewer 2, Comment 1**

‘This paper highlights interesting issues in patient-centred care.’

Author response: Thank you.

- **Reviewer 2, Comment 2**

‘Abstract: Sampling methods should be stated in the methods section.’

Author response: Our sampling methods have been added to the abstract. The abstract now reads:

‘This multi-site hospital ethnography was carried out in five ICUs in government hospitals in Bangladesh and India, selected using purposive sampling.’

- **Reviewer 2, Comment 3**

‘Abstract: A summary of recommendations should be stated in the conclusion section.’

Author response: A summary of the recommendations has been added. The conclusion of the abstract now reads:

‘Factors that promote person-centred care include access to ICU for families, support for family involvement in care delivery, clear communication with patients and families, good symptom management for patients, support for rehabilitation, and measures to address the physical, environmental and financial needs of the family.’

- **Reviewer 2, Comment 4**

‘Justification on why the study should be conducted in public facilities should be added.’

Author response: We have added a justification to the methods section. The manuscript now reads:

‘Government hospitals were chosen to allow a specific focus on public facilities, with the goal of producing study findings that are relevant to guide government policy.’

- **Reviewer 2, Comment 5**

‘Methods: Please explain the sampling methods of the five hospitals’

Author response: At your suggestion we have added an explanation of the sampling methods. The manuscript now reads:

‘A multisite hospital ethnography was carried out in five ICUs in hospitals in Bangladesh and India. The sites were selected using purposive sampling based on their funding model (all government hospitals) and their size (all large regional units). The distance between the sites was an additional factor influencing site selection in India to enable the same research team to access both units; this was not relevant in Bangladesh as the country is smaller. Government hospitals were chosen to allow a specific focus on public facilities, with the goal of producing study findings that are relevant to guide government policy. Large regional ICUs with bed numbers of 12 or above were chosen to make comparisons between the sites more meaningful.’

- **Reviewer 2, Comment 6**

- Please explain the sampling methods of the patients and care families

Author response: We have added an explanation of the sampling methods. The manuscript now reads:

‘Patients and family members who met these criteria were sampled consecutively.’

- **Reviewer 2, Comment 7**

- Please elaborate on the questions in the interview guideline

Author response: We have added the interview guide as a supplementary file.

- **Reviewer 2, Comment 8**

- Please explain the observation checklist and add it in the supplementary file.

Author response: The observations conducted for this study were unstructured and an observation checklist was not used.

- **Reviewer 2, Comment 9**

- Please describe the results of coding using thematic analysis

Author response: The results are presented according to the themes and subthemes that were generated during the thematic analysis. To address this point we have added an additional figure to the manuscript.

- **Reviewer 2, Comment 10**

- Did the author use data saturation to define the final number of interviews and observations? Please explain.

Author response: Thank you for the question. We did not use data saturation to define the number of interviews and observations. Instead, we made preliminary estimates of the numbers of interviews and duration of observations likely to be needed to answer our research questions using the concept of information power, using the approach described by Malterud et al. (Qual Health Res, 2016). We then revised these estimates during data collection by assessing the adequacy of the collected data to address our research questions. We have expanded our description of this process in the manuscript. It now reads:

‘The target number of interviews and the approximate duration of observations in each ICU was determined prior to the start of data collection using the concept of ‘information power’ (13). These estimates were revised during data collection based on regular appraisals of the adequacy of the data collected to address the research questions (14). Data adequacy was assessed during weekly team discussions. The final number of interviews conducted and the duration of observations in each site is given in table 1.’

- **Reviewer 2, Comment 11**

‘Results: Please put the initials of the informant in the quotation, for instance, a senior nurse 1, a senior nurse 2, doctor 1, etc. So that we can have insight that the quotations are coming from various informants.’

Author response: Added!

- **Reviewer 2, Comment 12**

'Discussion: Please discuss whether the findings can be influenced by cultural aspect of people in India and Bangladesh'

Author response: Thank you for this interesting question. It is very likely that cultural aspects in India and Bangladesh influence some of our findings. One example is the prominent role of family in both countries; this is likely to influence the care-giving behaviours we observed in the study. In the manuscript we discuss certain cultural aspects, including the veneration of doctors and the response to pain. Further exploration of the influence of culture could be an interesting focus of future work.

- **Reviewer 2, Comment 13**

'Please discuss about the generalisability of the findings in other context in low and middle income countries.'

Author response: We identified factors that influence patient and family experience that are important in Bangladesh and India specifically; these may be of relevance in comparable contexts. Without further study, we do not feel able to comment further on the generalisability of our findings.

The manuscript now reads:

'Many of our findings in this study align with evidence from other critical care settings (21). However, we have also identified additional factors that are important in Bangladesh and India specifically, and may be of relevance in comparable contexts.'

- **Reviewer 2, Comment 14**

'The article needs English editing.'

Author response: Thank you. We have reviewed the manuscript with this in mind.

- **Reviewer 3, Comment 1**

'The study demonstrated commendable strength in its comprehensive exploration of critically ill patients', families' and staffs' experiences in ICUs in Bangladesh and India. The incorporation of international evidence, practical recommendations, and understanding of resource constraints added value to the understanding of person-centered care in diverse healthcare settings. The study's robust methodology, including a reflexive thematic analysis and cross-team collaboration, contributed to its credibility and potential for real-world impact.'

Author response: Thank you.

- **Reviewer 3, Comment 2**

‘Study design section: It would be helpful to have a clear justification for why these specific hospitals in Bangladesh and India were chosen. Did they represent a diverse range of patient populations, geographical locations, or other relevant factors?’

Author response: Thank you for the suggestion. We have expanded this section.

The manuscript now reads:

‘The sites were selected using purposive sampling based on their funding model (all government hospitals) and their size (all large regional units). The distance between the sites was an additional factor influencing site selection in India to enable the same research team to access both units; this was not relevant in Bangladesh as the country is smaller. Government hospitals were chosen to allow a specific focus on public facilities, with the goal of producing study findings that are relevant to guide government policy. Large regional ICUs with bed numbers of 12 or above were chosen to make comparisons between the sites more meaningful.

All five ICUs treat a mix of medical and surgical adult patients. They all follow a predominantly closed ICU model, meaning the intensive care team is primarily responsible for patient care and decisions about who to admit. At the request of the centres involved, no further information regarding the sites will be provided to protect confidentiality. This was requested as a condition of participation, given the sensitive nature of the data collected and the perceived potential for reputational damage to the host institutions that could ensue.’

- **Reviewer 3, Comment 3**

‘Study design section: Rationale for Confidentiality. While confidentiality is mentioned, providing a brief rationale for why it's crucial in this study can add depth. For example, explain whether revealing hospital names could lead to potential biases or compromises in the research.’

Author response: Thank you for the suggestion. We have expanded our explanation as follows.

‘At the request of the centres involved, no further information regarding the sites will be provided to protect confidentiality. This was requested as a condition of participation, given the sensitive nature of the data being collected and the perceived potential for reputational damage to the host institutions that could ensue.’

- **Reviewer 3, Comment 4**

‘Observations: While it is mentioned that oral consent was obtained for observations, more details on the nature of the consent process, including how it was explained to participants and any potential challenges faced, would strengthen the transparency of the study.’

Author response:

We have updated our description of the consent process as follows:

‘Consent

Prior to starting data collection in each site, the research team held a series of meetings with the ICU staff to explain the purpose of the study. The meetings were staggered to allow staff working different shifts to attend. The content of the meeting was structured around the information on the participant information leaflet and attendees were given a copy of the participant information leaflet in a language of their choice. Staff were invited to ask questions during the meetings or to speak to a member of the research team privately afterwards; for several days after the meeting the researchers made themselves available in a private area of the ICU for this purpose. It was emphasised that involvement in the study was entirely optional. Two members of staff across the five sites initially declined to take part; both changed their mind and asked to get involved part way through the study.

After completing the preliminary steps of the consent process outlined above, verbal consent was obtained from staff at the start of each period of observation. Verbal consent was also obtained from family members when they were present during the observation. Although not the direct focus of the observations, patients were made aware of the presence and purpose of the research team whenever their condition allowed. This was not possible in many cases because they were unconscious or sedated.

Written consent was obtained prior to all interviews. Adapted versions of the participant information leaflet were used for patients and family members. During the consent process it was emphasized that the decision to participate or not would not influence the medical care they received.’

- **Reviewer 3, Comment 5**

‘The clinician researcher who continued clinical work during observations may face challenges in maintaining objectivity. Additional information on how potential biases associated with multitasking were addressed would enhance the credibility of the data.’

Author response: Thank you for highlighting this. We fully acknowledge the influence of this clinician researcher’s continuing clinical work on the data they collected. In the words of Braun and Clarke (Int J Transgend Health, 2023): ‘Reflexive thematic analysis is premised on the researcher always shaping their research; it will always be infused with their subjectivity, and they are never a neutral conduit, simply conveying a directly-accessed truth of participants’ experience.’ The positionality of the different members of the team was frequently discussed and reflected upon during data collection and analysis.

We have updated the manuscript as follows:

‘The researchers’ differing roles, degree of participation during observation, and positionality all influenced the data that was collected. Data collection was organised in such a way that observational data was often collected from the same event in tandem by two researchers with different professional backgrounds (one physician

and one social scientist) so these influences could be interrogated and were discussed frequently. The resulting insights added depth to the data.'

- **Reviewer 3, Comment 6**

'While patients were made aware of the research team's presence when their condition allowed, it would be beneficial to elaborate on how the team managed where patient conditions might have limited their understanding or ability to provide consent?'

Author response: We have revised the manuscript as follows:

'Although not the direct focus of the observations, patients were made aware of the presence and purpose of the research team whenever their condition allowed. This was not possible in many cases as they were unconscious or sedated.'

- **Reviewer 3, Comment 7**

'The varying degrees of participation among researchers during observations could introduce potential bias. Further clarification on how this variability was managed, ensuring consistency in data collection?'

Author response: Please see the response to reviewer 3, comment 5, above.

- **Reviewer 3, Comment 8**

'Limited Details on "Information Power": While the concept of "information power" is mentioned in determining the target number of interviews, more details on how this concept was practically applied, especially in terms of data saturation, would enhance the methodological transparency.

Author response: We did not use data saturation to define the number of interviews and observations. Instead we made preliminary estimates of the numbers of interviews and duration of observations likely to be needed to answer our research questions using the concept of information power, using the approach described by Malterud et al. (Qual Health Res, 2016). We then revised these estimates during data collection by assessing the adequacy of the collected data to address our research questions. We have expanded our description of this process in the manuscript. It now reads:

'The target number of interviews and the approximate duration of observations in each ICU was determined prior to the start of data collection using the concept of 'information power' (13). These estimates were revised during data collection based on regular appraisals of the adequacy of the data collected to address the research questions (14). Data adequacy was assessed during weekly team discussions. The final number of interviews conducted and the duration of observations in each site is given in table 1.'

- **Reviewer 3, Comment 9**

‘While it is mentioned that one researcher reviewed all transcripts and translations for quality control, additional details on the specific measures taken for quality control, such as inter-rater reliability checks?’

Author response: In Bangladesh, the initial transcription and translation were done by a small team of people but the same reviewer checked all transcriptions and translations. Since the same reviewer was checking them all, no additional inter-rater reliability checks were carried out.

- **Reviewer 3, Comment 10**

‘For interviews conducted in India, where bilingual interviewers translated directly, more details on the process of crosschecking and resolving translation queries could provide insights into the rigor of the translation process.’

Reviewer response: At your suggestion we have clarified this process.

The manuscript now reads:

‘In India, interviews conducted in Hindi or the local language were translated directly into English by the two trilingual interviewers, crosschecking with each other when there were Hindi translation queries. A third person was consulted for queries in the local language. It was possible to resolve all translation queries in this manner. The interviews conducted in English were transcribed only.’

- **Reviewer 3, Comment 11**

d. Data analysis:

‘While the coding process is mentioned briefly, providing more detail on how codes were developed, refined, and the criteria for inclusion/exclusion of codes would provide a clearer understanding of the analysis process.’

Reviewer response: In line with the reflexive thematic analysis approach, during the process of analysis there was no code book developed or explicit criteria for inclusion or exclusion of codes. Our data analysis was predominantly inductive, but themes that were relevant to the research questions were prioritised for inclusion. We have clarified this process in the manuscript as follows:

‘Once data collection was complete, RI examined the full dataset and highlighted data that broadly pertained to patient or family experience. These data were then open-coded based on semantic meaning. Preliminary themes were created from the coded data in a recursive manner, going back to refine the coding where needed. As the analysis progressed, a more deductive approach was used to ensure that the themes and subthemes being generated were of relevance to the research questions. During the later stages of analysis, research team members who were not directly involved in data collection reviewed the preliminary themes to sense-check and probe the theme descriptions, to enhance reflexivity and analytical depth.’

- **Reviewer 3, Comment 12**

'The term "recursive manner" is used in describing the generation of preliminary themes. Further elaboration on how this recursive process was conducted would enhance the clarity.'

Reviewer response: We have explained this in more detail as follows:

'Preliminary themes were generated from the codes and the coded data in a recursive manner, going back to refine the coding where needed.'

- **Reviewer 3, Comment 13**

3. It would be beneficial to clarify the role of the clinician researcher who continued to work clinically during observations in the analysis phase. How was potential bias managed in this context?

Author response: Please see the response to reviewer 3, comment 5 above.

- **Reviewer 3, Comment 14**

4. While the involvement of team members in reviewing preliminary themes is mentioned, providing more detail on specific instances of reflexivity and how it enhanced analytical depth would add depth to the methodology.

Author response: Team members with different professional/research backgrounds (intensive care medicine, social science and implementation research) were involved in regular debriefing meetings (weekly during data collection). During these meetings researchers from different sites would share and compare findings of importance. The discussions would inform subsequent data collection. After data collection, additional discussions were held with all team members (including those not directly involved in the ongoing debriefs) to discuss the preliminary analysis. We have added some text to the data analysis section that outlines this process.

- **Reviewer 3, Comment 15**

Minor issue: Study design section: The study's purpose, research questions, or objectives should be stated before the method section and not within the study design.

Author response: the research questions are now before the study design section.

- **Reviewer 3, Comment 16**

Minor issue: In addition to this, I recommend considering the inclusion of the semi-structured interview questionnaire as a supplementary material or in the main manuscript if space allowed. This addition would contribute to the transparency of the study and provide understanding of the specific questions posed during the interviews. It can also enhance the reproducibility of the research and allow future studies to build upon or replicate the methodology. Additionally, it enables the researchers to evaluate the alignment between research questions, interview questions, and the study's objectives.

Author response: we have now included the interview guides in an appendix.

- **Reviewer 3, Comment 17**

c. The discussion section is commendable for its clear identification of key areas influencing ICU experiences in Bangladesh and India. The practical recommendations derived from the study findings are actionable and demonstrate a thoughtful consideration of resource constraints, contributing to the pragmatic impact of the research. I recommend to consider delving further into doctor-patient-family dynamics to enhance the depth.

Author response: many thanks for this complementary remark. Given the current length of the manuscript (at over 10 000 words) we feel that there is insufficient space to extend discussion of the doctor-patient-family dynamics in this paper. The topic will be examined in more detail in a later publication.

We have attached two versions of the revised manuscript, one clean and one with tracked changes. We greatly appreciate the time and effort that you and the reviewers have given to providing feedback and are grateful for the insightful comments and suggested improvements to our paper.

Your sincerely,

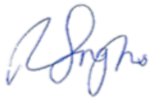

Rebecca Inglis
